# Practical Handbook of the Diseases of the Eye

**Published:** 1898-06

**Authors:** 


**Practical Handbook of the Diseases of the Eye.** By D. Chalmers Watson, M.B. Pp. xii., 236. Edinburgh: William F. Clay. 1897.

The author has not attempted to add another to the long list of large text-books of which the already overtaxed student has to make choice.

Now, however, that the study of ophthalmology has become a necessary item in the medical curriculum, it is only fitting that some short account of the subject should be placed within reach of those wishing to avail themselves of the clinical material to be found in the eye department of a large hospital. It is not necessary, nor indeed is it possible, for every medical man to make himself familiar with all those ills that the eye is heir to; but he should, at least, be able to differentiate between such maladies as iritis and glaucoma, and so avoid the terrible consequences that their confusion entails.

The subject matter of this work is very sound, and is simply arranged. The diagrams and plates are clear and expressive, and the work can be confidently recommended as a most satisfactory student's handbook. It contains all that is necessary for a very fair knowledge of ophthalmic work, whilst its size, lucidity, and usefulness should secure for it general acceptance.

